# Mediterranean Gluten-Free Diet: Is It a Fair Bet for the Treatment of Gluten-Related Disorders?

**DOI:** 10.3389/fnut.2020.583981

**Published:** 2020-12-02

**Authors:** Karla A. Bascuñán, Luca Elli, Maurizio Vecchi, Alice Scricciolo, Federica Mascaretti, Maria Parisi, Luisa Doneda, Vincenza Lombardo, Magdalena Araya, Leda Roncoroni

**Affiliations:** ^1^Center for Prevention and Diagnosis of Celiac Disease, Gastroenterology and Endoscopy Unit, Fondazione Istituto di Ricovero a Carattere Scientifico (IRCCS) Ca' Granda Ospedale Maggiore Policlinico, Milan, Italy; ^2^Department of Nutrition, School of Medicine, University of Chile, Santiago, Chile; ^3^Department of Pathophysiology and Transplantation, University of Milan, Milan, Italy; ^4^General Surgery Unit, Fondazione Istituto di Ricovero a Carattere Scientifico (IRCCS) Ca' Granda Ospedale Maggiore Policlinico, Milan, Italy; ^5^Department of Biomedical, Surgical, and Dental Sciences, University of Milan, Milan, Italy; ^6^Institute of Nutrition and Food Technology, Instituto de Nutrición y Tecnología de los Alimentos (INTA), University of Chile, Santiago, Chile

**Keywords:** gluten-free diet, Mediterranean diet, food pyramid, cereals, pseudocereals

## Abstract

Gluten-free diet (GFD) is the current treatment of gluten-related disorders. It eliminates wheat, barley, and rye, while the exclusion of oats is still under debate. GFD is based on a combination of naturally gluten-free foods and gluten-free substitutes of cereal-based foods. Although effective as treatment of gluten-related disorders, today there is concern about how to improve GFD's nutritional quality, to make it not only gluten-free, but also healthy. The “Mediterranean diet” (MedD) refers to the dietary pattern and eating habits typical of populations living in the Mediterranean basin, which have been associated with low prevalence of several diet-related pathologies. Here we present a narrative review of the current knowledge about GFD and MedD, their characteristics and central food components. Based on the Mediterranean diet pyramid developed by the Italian pediatric society, we propose a combination between the MedD and the GFD, an attractive alternative to reach a gluten-free state that at the same time is healthy, with a clear benefit to those who practice it.

## Introduction

Gluten related disorders share in common that they are triggered by gluten ingestion. They are a changing group of conditions, including celiac disease (CD), wheat allergy (WA), and non-celiac gluten sensitivity (NCGS) (1). Although mediated by different pathogenic pathways, their clinical manifestations may be similar (2), posing difficulties at the time of diagnosis (3). Their only effective treatment is a gluten-free diet (GFD), which eliminates wheat, rye, and barley consumption. GFD consists of a combination of naturally gluten-free foods (GFF) and gluten-free substitutes prepared with a variety of gluten-free foods and cereals (4). Although highly effective, GFD requires supervision by a trained professional, who must educate the patient and manage the diet, because this is often poor in fiber and vitamins and high in lipid and sugar content (5). In fact, excessive weight and obesity can also be a concern in celiac patients following a GFD, due to the rich-energy gluten- free foods/products commonly eaten (6). Currently available gluten-free products are often low in protein, with high fat and salt content. Higher levels of dietary fiber and lower sugar content have been recently reported (7). High consumption of rich-energy and rich-fat foods to compensate for dietary restriction has been reported in both adults and children with CD (8). There is concern today that the GFD may contribute to a microbiota signature (9) and cardiometabolic risk (obesity, dyslipidemia, insulin resistance, and metabolic syndrome) (8, 10, 11). However, these risks are still not clear, a systematic review and meta-analysis concluded that CD was associated with a moderately increased cardiovascular risk (CVD), with limited evidence available (12).

The Mediterranean diet (MedD) is defined as the dietary pattern of people inhabiting the Mediterranean region. It is characterized by relatively high consumption of cereals, vegetables, legumes, fruits, nuts, fish, and olive oil, with moderate amounts of meat, milk and derivatives, sugars, and wine (13). This diet has proved successful in promoting health throughout life, contributing to the prevention of non-communicable chronic diseases (NCCD) (14). We therefore propose to analyze whether a MedD may be useful for those who must avoid gluten intake. We here review the MedD and GFD and, applying the concept of the food pyramid (15) used to teach the food groups that one should consume daily to remain healthy, we propose a gluten-free MedD pyramid.

## Gluten-Free Diet

Wheat has been essential to the diet since the first agricultural revolution in 10.000 BC. Gluten is contained in wheat, barley, and rye (16). Is a cohesive and visco-elastic material present in the alcohol-soluble fraction of gliadin and glutenins, widely consumed in customary Western diets (17, 18). The food industry extensively uses gluten due to its capacity to preserve the air in the protein matrices, improving the baking process, and enhancing properties of the processed foods (19, 20). Still, today GFD is also the treatment of patients with other gluten-related disorders, including NCGS and WA (18, 21). Replacing gluten-containing cereals is often technically challenging, and GFF often contain more lipids or more carbohydrate. Unless education and training are provided to the celiac patient, his/her choice will include a proportion of unhealthy foods, and additional health risks will be added (22).

## Gluten Substitutes in GFD

The only relevant change in GFD refers to the type of cereals to be included. This does not imply necessarily deterioration of the nutritional food value, because today, several cereals and pseudocereals do not contain gluten that can substitute wheat gluten (Table 1) (23).

**Table 1:** Nutritional characteristics of gluten-free alternative cereals.

**Cereals**	**Nutritional characteristics**
Pseudo-cereals Buckwheat (*Fagopyrum esculentum*)—Central Asia Amaranth (*Amaranthus cruentus*)—South America Quinoa (*Chenopodium quinoa* Willd.)—South America Chia (*Salvia hispanica*)—Central America	• Source of antioxidants: carotenoids in quinoa seeds, mainly represented by lutein, zeaxanthin, and carotene (24); Chlorogenic acid, caffeic acid, myricetin, quercetin, and kaempferol contained in chia seeds (25); Polyphenols such as quercetin, apigenin, and luteolin contained in buckwheat (26) • The protein content of quinoa, amaranth, and buckwheat, has been reported to be 12.0–18.9% and essential amino acids, particularly, cysteine and methionine, are known to be higher than rice and maize (27) • High amount of dietary fiber (28) • Quinoa has the highest fat content between 2.0 and 9.5%, rich in essential polyunsaturated fatty acids such as linoleic and α-linolenic (28) • The presence of some “anti-nutritional factor” reduces nutritional value, interfering on digestibility, absorption, or utilization of nutrients. The antinutritional factors in the quinoa seed are: saponins, phytic acid, tannins, nitrates, oxalates, and trypsin inhibitors (28)
Teff (*Eragrostis tef*)—Ethiopia and Eritrea	The flour is rich in fiber (3.0/100 g) • The germ and bran are consumed along with the endosperm • A source of bioactive compounds such as polyphenols • Protein content is 10.5–12.8% • Rich in minerals such as calcium, zinc, magnesium, iron, phosphorous, and copper • Contains good levels of vitamin C, niacin, vitamin A, riboflavin, and thiamine
Sorghum [*Sorghum bicolor* (L.) *Moench*]—North-eastern Africa	• High presence of fibers • Contains bioactive compounds: proanthocyanidins, 3-deoxyanthocyanidins, and flavones
Rice (*Oryza sativa* L.)—Asia	• The health benefits of whole grain derive mainly from one of its major constituents, i.e., the polyphenols
Maize (29) (*Zea mays*)—*M*exico	• Provides many of the B complex vitamins, essential minerals, and fiber • Lacks vitamin B_12_, vitamin C, and is a poor source of calcium, folate, and iron
Oat (*Avena sativa*)—England, France, Poland, Germany, and Russia	• Starch is the major component • High amounts of soluble food fiber in particular beta-glucans (30) • Grains contain natural antioxidants: tocols (tocopherols and tocotrienols) and vitamin E (31)

### Gluten-Free Pseudocereals

“Pseudocereals” is a non-botanical term that refers to dicotyledonous plants, while cereals refer to gramineae (Table 1). Pseudo-cereals are buckwheat, amaranth, quinoa, and chia. Chia (*Salvia hispanica*) originated in Mexico and Guatemala; Quinoa (*Chenopodium quinoa* Willd) and Amaranth (*Amaranthus cruentus*) are grown in South America and Buckwheat, originally from Central Asia, is now grown in Central and Eastern Europe (32). Pseudo-cereals are gluten-free and a suitable option to enhance the nutritional value of GFF. They are high in fiber, have good quality proteins, and unsaturated fatty acids (33). We briefly summarize their main characteristics in the next paragraphs.

#### Antioxidants

Pseudo-cereals are rich in antioxidants, mainly phenol functional groups, including flavonoids and phenolic acids, carotenoids, and tocols. They prevent oxidation and reactive oxygen species generation. Free radicals, generally in the form of reactive oxygen species, are by-products of cellular redox processes; in low concentrations, antioxidants have beneficial effects on cellular responses and immune function. Instead, at high levels, they cause oxidative stress by modifying cell structure and organelles. Today, it is generally agreed that oxidative stress plays a significant role in the development of chronic and degenerative diseases (34–38).

#### Proteins

Among pseudo-cereals, quinoa, amaranth, and buckwheat are non-conventional sources of good quality proteins. Although grown for thousands of years, they are not widely used due to their high price (39). Quinoa and buckwheat contain all essential amino acids and are also rich in sulfur amino acids and lysine, unlike other cereals, which are deficient in lysine (40).

#### Carbohydrates and Dietary Fiber

Quinoa has a high content of starch (41) that form semi-crystalline structures referred to as “starch granules” (42) and a small percentage of sugars (~3% refined sugar). All pseudo-cereals contain a high amount of dietary fiber, improve digestibility, and facilitates other nutrients absorption process in the large intestine (40). Besides, the consumption of dietary fiber is associated with higher post-meal satiety and decreased subsequent hunger (43).

#### Lipids

Quinoa has a significant lipid fraction, especially rich in essential polyunsaturated fatty acids. In recent decades, polyunsaturated fatty acids have gained importance because of their positive effects on CVD, prostaglandin metabolism, insulin sensitivity, the immune system, and cell membrane function (40). Chia is also an excellent source of mega-3 fatty acid (about 65% of the oil content), especially α-linolenic acid (43).

#### Micro-Nutrients

Quinoa is a good source of vitamin E, has high levels of thiamine, folic acid, and vitamin C. When compared to other cereals, quinoa is a good source of iron. However, its availability is limited by the presence of saponins and phytic acid. Amaranth and quinoa are good sources of calcium, iron, and magnesium; this is important because GFF are usually deficient in these minerals. Calcium, magnesium, and iron are the main mineral deficiencies in gluten-free products. Increasing calcium content is relevant for celiac patients because they frequently suffer osteopenia and osteoporosis (40). Chia biological value is better than that of other cereals, and also, its content of calcium, magnesium, and potassium is higher than in milk (43). Buckwheat is an excellent source of manganese, copper, magnesium, phosphorus, and vitamins (27).

Despite the known nutritional benefits of pseudo-cereals, several studies indicate that their use in GFF production is still scarce (23, 32, 33, 39, 42, 44, 45). A Brazilian study analyzed the ingredients of GFF vs. their gluten-containing counterparts. It showed that sugar was the most frequent ingredient in both types of products' labels, along with salt and sodium. No GFF contained pseudo-cereals (24). The main ingredients were limited to rice, corn, soy, potato, and cassava. This latter is a perennial shrub of the Euphorbiaceae family, native to central South America, and currently cultivated in most tropical and sub-tropical areas of the American continent. It is valued for its roots rich in starches of high nutritional value (25). Do Nascimento et al. (24) analyzed the components used as substitutes of wheat flour in GFF in Brazil and found that these were mostly unfortified and refined (26, 46). This resulted in foods with low levels of B complex vitamins, iron, folate, and dietary fiber as compared with their gluten-containing counterparts. In Chile, a study that evaluated the “basic family basket” of GFF, described low protein content in comparison with the respective gluten-containing counterparts (47). Authors also found that while gluten-containing bread and cereals were mainly based on wheat, their corresponding GFF were based on rice, corn, cassava, and potato starch, all ingredients with low protein content and poorer nutritional quality (28, 48).

### Gluten-Free Cereals

- Teff (*Eragrostis tef* ) is an annual crop of the Poaceae (grass) family, native of Ethiopia and Eritrea. Teff grain is used as ingredient of foods and beverages because of its better nutritional properties in comparison with other grains, like wheat, barley, and maize. Teff's grain size is small and usually is eaten as wholegrain, therefore providing a good amount of fiber. It is also a good source of polyphenols. The comparative study of two teff varieties showed that their protein content was 10.5–12.8%, which is higher than most other cereals (49, 50). It is also rich in minerals (calcium, zinc, magnesium, iron, phosphorous, and copper) and vitamins (C, A, niacin, riboflavin, and thiamine) (50).
- Sorghum [*Sorghum bicolor* (L.) Moench] originated in northeastern Africa. It contains high levels of fiber and bio-active compounds, including proanthocyanidins, 3-deoxyanthocyanidins, and flavones. There is promising evidence showing that it inhibits cancer cell growth *in vitro*, and also, it has anti-inflammatory effects in animal models (51).

- Rice (*Oryza sativa* L.) is a GFF food consumed globally. While white rice is consumed worldwide, Asian populations also consume pigmented rice, known as black, purple, red, and brown rice. Black rice has a high concentration of tocopherols, oryzanols, polyphenols, B vitamins, and fiber. Black rice is particularly rich in anthocyanins, water-soluble flavonoids with antioxidant properties. Wholegrain rice is the unpolished version of grains containing the germ, bran, and endosperm. The health benefits of whole grain rice derive mainly from one of its polyphenols (52).
- Maize (*Zea mays*) is thought to be native of Mexico. It contains low amounts of calcium, folate, and non-heme iron. Fortification of maize flour and cornmeal with iron, other vitamins, and minerals is used to improve micronutrient intake and prevent iron deficiency in celiac patients (29).

- Oat (*Avena sativa*) originated in Europe and is now grown worldwide. Its primary component is starch (60% of total dry weight), which digests slowly due to the presence of high amounts of fiber and oil that delays stomach emptying and favors digestion. As a result, a gradual supply of glucose, yielding low glycemic index, and prolonged satiety can be reached. Oat groats protein content is relatively high (15–20% by weight), and its digestibility (90%) is better in comparison to rice and corn protein (30). Oat contains tocopherols, tocotrienols, and vitamin E, which help reducing serum cholesterol and inhibit cell growth in certain cancers (31, 53), properties mainly attributed to the high content of oat-specific beta-glucans. Oat fibers increase the fecal bulk and have a positive effect on the intestinal microbiome. Less known bioactive components are the avenanthramides (phenolic amine conjugates); they have anti-inflammatory properties through the suppression of prostaglandin E2. Oats also suppresses vascular smooth muscle cell proliferation, a process known to be a contributing factor in the development of atherosclerosis (30). Yet, the effects of including oats in GFD are still unclear. Oats are most often contaminated with wheat, and therefore, the presence of gluten in oat-containing GFF must be assessed case by case (54–58). A Canadian Position study recommends consuming only those products included in the National Register of GFF of the Ministry of Health (59). Concern also rises from evidence suggesting that some oat varieties might have direct toxicity in CD. This is based in *in-vitro* studies that show that some oat varieties activate peripheral blood lymphocytes obtained from coeliac patients; the hypothesis is that the same phenomena could occur in duodenal mucosa of celiac persons, triggering gluten-dependent inflammation (60). At present, it is widely agreed that to introduce oats in GFD's patients, these must be in complete remission and receiving GFD (that excludes oats) for at least 6 months (54–56, 59).

## The Mediterranean Diet and Its Origins

The MedD originated in the Mediterranean basin, where many populations in human history blended and developed. This region was a meeting place for people coming from different cultures, customs, languages, religions, eating habits (61). Over the centuries, the MedD incorporated foods from different cultures, like vineyards and olive trees (62). The discovery of America led to acquiring new culinary traditions and new food products, like tomatoes, peppers, chili peppers, corn, beans, and potatoes. Tomato was initially described as “exotic curiosities;” interestingly, they were the first red vegetable that enriched the Mediterranean food basket, subsequently becoming a symbol of the Mediterranean cuisine (61).

### Early Studies

Since the mid-twentieth century, the available evidence showed that MedD diet protected against CVD and heart (CHD) diseases and was associated with lower all-cause mortality rates (63). In the early 1900s, De Langen noticed that Indonesian natives had lower serum cholesterol and lower risk of developing angina pectoris in comparison to Dutch people (63). In the 1950s, an epidemiologic study in Crete revealed that Cretans' diet contained more plant foods together with lower milk, meat, and fish consumption, compared to USA feeding patterns. Remarkably, meals were described to be “swimming” in olive oil and that this would “preserve the nutritive value of the food rather well” (64, 65). Later, Keys analyzed Italian data and observed that both blood cholesterol levels and frequency of heart attacks in the general local population were lower than in the more affluent groups. These and other studies contributed to the hypothesis that the prevalence, incidence, and mortality caused by CVD and CHD differ between different populations. Also, eating habits and other conditions can explain these differences, and that CVD and CHD episodes can be predicted based on individual (dietary) characteristics.

Today, the MedD does not only consist of a food consumption pattern, it also includes a pro-active lifestyle with regular physical exercise (62). MedD became part of the UNESCO Intangible Cultural Heritage list in 2010. Results of the 2003–2011 PREDIMED (Prevención con Dieta Mediterránea) study further reinforced the value of MedD for health promotion and proposed it as a model to follow globally (65).

The MedD has been inversely associated with several diseases, including some extensively investigated like CVD and cancer, alongside with associations with mental health, immunity, and quality of life (66). MedD has been described to change taxa enrichment in the intestinal microbiota, and adherence to the diet was positively associated with markers of lower frailty and improved cognitive functions in the elderly after 1-year intervention (67). Also, some of the microbiome changes were negatively associated with inflammatory markers, including C-reactive protein and interleukin-17. As stated by the authors, these findings suggest the possibility of modulating the gut microbiota by means of the diet, favoring healthy aging (67). A systematic review of five RCTs (68) on the effect of MedD in cognition and brain morphology and function showed that the data are mostly non-significant, however, the significant improvements in the cognitive domain compounds, in the more robust design study warrant further investigation (68). On the other hand, some reports suggest that adoption of the MedD could counteract the effects of several inflammatory components, decreasing the secretion of circulating and cellular biomarkers involved in the atherosclerotic process (69). The study of potential mechanisms that could explain such associations suggest that it is not only the nutrient dietary content, but the interaction and combination of foods, nutrients in food, non-nutritive substances, cooking techniques and lifestyle habits that make the MedD a potential tool to prevent and treat these situations (70). The scientific evidence that supports the practice of MedD for the prevention and treatment of a wide variety of conditions, reinforces the idea of promoting its use in patients who must follow restrictive diets, favoring and providing the basis for a healthier diet that can improve the quality of life of CD patients.

## The Mediterranean Diet, Functional Foods, and Grains

Some foods such as phytochemicals, sulfur-containing compounds, carotenoids, monoterpenes, phytosterols, and different polyphenols. Today they classify as “nutraceuticals,” the hybrid concept that mixes pharmaceuticals and nutrients (71, 72). Functional foods are those foods containing specific “nutraceuticals,” which enhance health or reduce the chance of disease when regularly eaten. Vegetables are relevant components of the Mediterranean diet, rich in flavonoids and phytosterols. Fruits are good sources of terpenes, flavonoids, minerals, and vitamins, which protect against oxidative stress. Extra virgin olive oil has high concentrations of monosaturated fatty acid and phytochemicals. Recent studies show that olive oil plays a role in bone mineralization, reducing the risk of osteoporosis. Herbs, spices, onions, and garlic, which are widely used in the Mediterranean diet as condiments, contain flavonoids. Whole grains provide dietary fiber, antioxidants, resistant starch, phytoestrogens, and essential micro-nutrients. In the grain-refining process, most of the bran and some of the germ is removed, resulting in loss of dietary fiber and some vitamins, minerals, lignans, phytoestrogens, phenolic compounds, and phytic acid (Table 1).

In practical terms, MedD includes 3–5 daily servings of any of the following: wheat, corn, spelt, barley, sorghum, millet, buckwheat, quinoa, or rice. Whole grains can be consumed as wholemeal flour, used to produce bread, pasta, and other foods. A recent systematic review investigated the relationship between the intake of whole grains and CVD, concluding that there is a significant reduction of cardiovascular risk, stroke, CHD, type-2 diabetes, and obesity, for each 90 g/day (3 servings) increase of whole-grain intake (73, 74).

How foods are prepared is most relevant in MedD. When cooking tomatoes, for example, the addition of olive oil increases the absorption of lycopene carotenoids, improving its bio-availability and antioxidant effect. Another interesting concept is that it is not that a single nutrient yields a beneficial effect; it is the whole meal and dietary pattern that achieves the results described for the MedD (71, 72).

The MedD is not just a food model but a mixture of art, science, habits, and traditions (including crops, harvesting, fishing, conservation, processing, and preparation of foods), all representing an integral part of the cultural heritage in the Mediterranean basin (75). It has incorporated lifestyle and cultural elements, which are located outside the pyramid. These include frugality, friendliness, traditions, sustainability, seasonality, and local foods. A recent addition is an adequate rest, which consists of a short nap during the day and at least 30 min daily physical exercise, both essential for a healthy lifestyle (76).

## The GFD-MedD Pyramid

In 1992, the US Department of Agriculture developed nutritional recommendations for the population in the form of a pyramid, and shortly after, the MedD pyramid was designed as a way to emphasize the same concepts in the context of the Mediterranean dietary habits (76, 77). The Italian Pediatric Society later developed the “trans-cultural food pyramid” to teach healthy food choices to children. This latter pyramid provides the basic notions of a balanced diet, incorporating foods from Asian, African, and American cultures. Its use has now extended to adults and is becoming a relevant tool to improve MedD adherence in a multi-ethnic society. The trans-cultural pyramid includes buckwheat, amaranth, quinoa, and chia, all of them gluten-free and reasonable alternatives for celiac patients, who otherwise restrict their carbohydrate sources to rice, potatoes, and corn.

**Figure 1:**
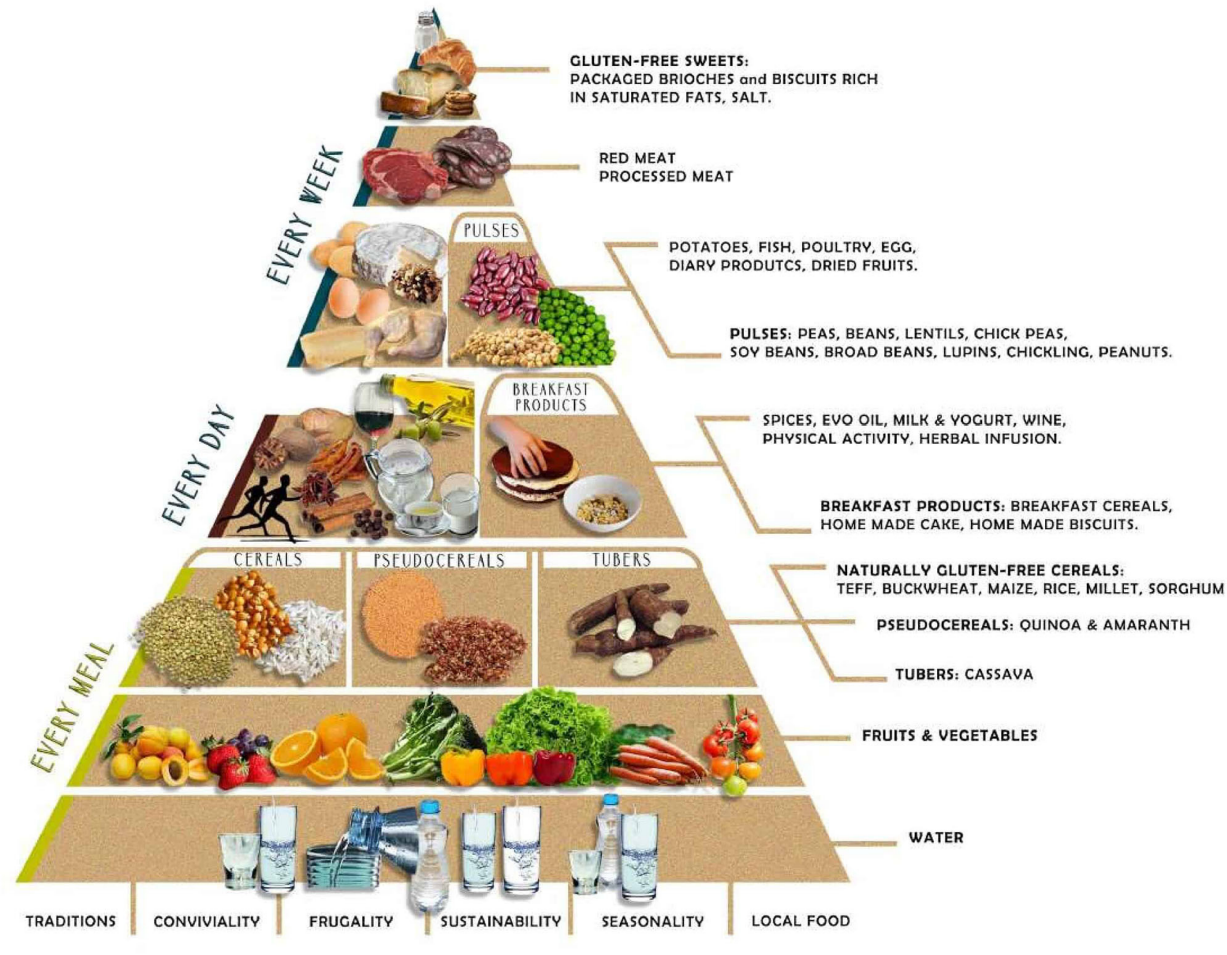
The Mediterranean GFD pyramid: a gluten-free lifestyle. A new graphic representation of the food pyramid based on the MedD is conceived, where coexistence with the GFD is possible. The Mediterranean GFD pyramid gathers updated recommendations considering lifestyle, dietary, sociocultural, environmental, and health challenges of individuals who follow a GFD in the context of healthy eating based on the MedD. MedD, Mediterranean diet; GFD, gluten-free diet.

GFD and MedD can be integrated into a Mediterranean-GFD pyramid (Figure 1). On the basis, water, fruit, vegetables, and cereals have a major role. The pyramid offers a variety of gluten-free cereals, pseudo-cereals, and tubers (cassava). This lowest section shows foods that are consumed every day; homemade breakfast preparations should be preferred, which may add spices, extra-virgin olive oil, milk, and yogurt, wine, and herbal infusions. After, are included those foods to be consumed weekly, like legumes, dried fruit, potatoes, and animal foods. In higher levels, red meat, and processed meat should be consumed less frequently, while white meats have less restriction. Gluten-free desserts should be only sporadically consumed and in small portions, because they are rich in simple sugars. Finally, it is worth considering that manufactured and naturally gluten-free products are not alike. The former tends to contain more saturated fats, sugar, and additives.

In summary, although large population studies are still lacking, this review provides robust arguments to propose the new GFD-MedD pyramid for those who must follow GFD, which will help making the diet not only gluten-free but also will make sure that it remains healthy. The Mediterranean-GFD Pyramid favors the use of raw materials and minimally processed GFF. Although wheat has a paramount position in the western diet it can be replaced by other cereals, some of which have improved nutritional characteristics.

## Author Contributions

KB, LE, AS, FM, MP, and LR wrote the paragraphs on GFD and cereals. LE, MV, VL, LD, and MA wrote the paragraphs on origins of MedD and functional foods. KB, LE, and LR described grains properties and the new MedD pyramid. KB, LR, and MA supervised the manuscript. All authors approved the final version. All authors contributed to the article and approved the submitted version.

## Conflict of Interest

LE is a member of the Dr. Schaer Institute scientific board. LE and LR are inventors of a patented gluten challenge test for NCGS. The remaining authors declare that the research was conducted in the absence of any commercial or financial relationships that could be construed as a potential conflict of interest. The handling Editor declared a past co-authorship with one of the authors MV.
